# Passive versus active service delivery: Comparing the effects of two parenting interventions on early cognitive development in rural China

**DOI:** 10.1016/j.worlddev.2021.105686

**Published:** 2022-01

**Authors:** Sean Sylvia, Renfu Luo, Jingdong Zhong, Sarah-Eve Dill, Alexis Medina, Scott Rozelle

**Affiliations:** a Gillings School of Global Public Health and Carolina Population Center, 1101-D McGavran-Greenberg Bldg., University of North Carolina at Chapel Hill, Chapel Hill, NC 27599, USA; b China Center for Agricultural Policy, School of Advanced Agricultural Science, 409 Wangkezhen Bldg., Peking University, No. 5 Yiheyuan, Haidian District, Beijing, China; c School of Economics, Peking University, No. 5 Yiheyuan, Haidian District, Beijing, China; d Rural Education Action Program, Freeman Spogli Institute for International Studies, 616 Jane Stanford Way, Stanford University, Stanford, CA, USA

**Keywords:** Center-based parenting intervention, Home-based parenting intervention, Early cognitive development, Randomized controlled trial, Program participation

## Abstract

We present the results of a cluster-randomized controlled trial that evaluates the effects of a free, center-based parenting intervention on early cognitive development and parenting practices in 100 rural villages in China. We then compare these effects to a previous trial of a home-based intervention conducted in the same region, using the same parenting curriculum and public service system, accounting for potential differences between the studies. We find that the center-based intervention did not have a significant impact on child development outcomes, but did lead to increases in the material investments, time investments, and parenting skills of caregivers. The average impact of the center-based intervention on child skills and investments in children was significantly smaller than the home-visiting intervention. Analysis of the possible mechanisms suggests that the difference in effects was driven primarily by different patterns of selection into program participation.

## Highlights

• It is unclear that what delivery model of early childhood interventions may be most effective in developing countries.
• We evaluate the effects of a center-based parenting intervention on early development and parenting practices in rural China.
• We compare the effects to a home-based intervention using the same curriculum and public service system in the same region.
• We find the home-visiting intervention was more effective than the center-based parenting intervention on child outcomes.
• The difference in effects appears to be driven by different patterns of compliance.

## 1 Introduction

Early childhood development (ECD) is central to the future of low- and middle-income countries (LMICs). Although child mortality rates in many LMICs have decreased dramatically in recent decades, approximately 250 million children under five years old in LMICs remain at risk of not reaching their developmental potential (Black et al., 2017). Because developmental outcomes in early childhood are critical to adult outcomes, including labor market returns (Gertler et al., 2014, Heckman et al., 2010, Huggett et al., 2011), health status (Campbell et al., 2014, Heckman, 2007), and social mobility (Heckman & Mosso, 2014), early developmental delays can have significant negative effects on later quality of life. At a macro level, widespread developmental delays can inhibit LMICs from raising human capital, which has been shown to be critical for sustaining long-term economic growth and development (Li, Loyalka, Rozelle, & Wu, 2017).

In light of this concern, a growing body of research has provided strong theoretical and empirical support for targeted ECD intervention programs that train caregivers in stimulating parenting practices. Cunha and Heckman, 2007, Cunha and Heckman, 2008, Cunha and Heckman, 2009, Cunha et al., 2010 have established a framework that shows that early parenting interventions can effectively boost the skills development of disadvantaged children and that parental investments in the earliest stages of life can effectively increase the impacts of later-stage investments. Empirical studies have further shown that parenting programs that target caregivers of young children can have meaningful effects on early skills. A recent meta-analysis of 21 randomized controlled trials (RCTs) of parenting interventions for children aged 0–3 conducted in LMICs since 2000 have found that parenting programs improved child cognitive development by 0.42 standard deviations (*SD*), on average (Aboud & Yousafzai, 2015).

Although past research has demonstrated the positive and significant impacts of parental training interventions on ECD outcomes, the question remains as to how to effectively deliver parenting intervention programs at scale. Building new infrastructure and employing new workers to deliver parenting interventions can be costly, particularly for LMICs that face more stringent resource constraints (Richter et al., 2017). As an alternative, international organizations (e.g., World Bank, United Nations, World Health Organization) have proposed that LMICs integrate ECD interventions into existing public infrastructure and public service systems (Chan, 2013, Richter et al., 2017).

To date, few parenting interventions in LMICs have been upscaled for widespread delivery. Among studies of potentially scalable parenting interventions, the interventions have relied mainly on one of two delivery models: one-on-one delivery or group-based delivery (Abdul Latif Jameel Poverty Action Lab [J-PAL], 2019). One-on-one parenting interventions typically involve regular visits from parenting trainers who conduct individual parenting lessons in the home, whereas group-based interventions require caregivers to bring their children to a central location to participate in group parenting training sessions. Several mechanisms may drive differences in the effectiveness of the two models, and it remains unclear which model may be more effective and cost-effective in different contexts (J-PAL, 2019). A third possible format is center-based parenting interventions, which can offer both one-on-one parenting training sessions and structured group activities in a central location, as well as provide a space for caregivers and children to engage in unstructured play. To date, there have been no studies that directly compare different delivery formats for parenting interventions. Although one-on-one and group-based models of parenting interventions have been evaluated independently (Grantham-McGregor et al., 2020, Walker et al., 2015), as well as evaluated together within the same intervention (e.g., Attanasio et al., 2018), past studies have been conducted by different research teams, using different curriculums, intervention, and outcome measures in different regions, complicating comparison between delivery models.

To address this gap, the goal of this study is to evaluate the effects of a free, center-based parenting intervention on ECD outcomes and parenting practices in rural China. We then compare the effects of the center-based delivery model with the effects of a home-based intervention previously conducted in the same region of rural China, using the same parenting curriculum and public service system. To implement the center-based delivery model, we worked with China’s National Health Commission (NHC) to conduct a large-scale, cluster-RCT of a center-based parenting intervention in 100 villages in an underdeveloped rural area in northwestern China. This center-based parenting program was implemented by the same research team in the same target area as a home-based program evaluated by Sylvia, Warrinnier, Renfu, Yue, Attanasio, Medina, and Rozelle (2018), used the same curriculum and public service system (NHC) to deliver the intervention, and measured the same outcomes, using similar instruments. Given these similarities, this comparison provides the best available comparative effects of these models absent a costly trial providing a direct head-to-head comparison. Our study compares the program treatment effects on child skill development and secondary parenting outcomes (caregiver investment and parenting skills) and examines potential sources of differential impacts between the two interventions.

Both delivery models are viable in rural China, where there is a need for scalable parental training interventions. Like other LMICs, China is facing widespread early developmental delays, with nearly half (49%) of rural children aged 0–3 years as exhibiting cognitive delay (Bai et al., 2019, Luo et al., 2017, Wang et al., 2019, Yue et al., 2017). At the same time, China has a large public infrastructure and abundant human resources, which can be leveraged to implement parenting interventions at scale should the government decide that this is a priority. Due to large-scale rural-to-urban labor migration and other demographic trends, there is a large number of disused schoolhouses, cultural centers, and office spaces in rural areas that can be repurposed for ECD centers. China also has one of the largest health bureaucracies in the world, NHC, available to implement parenting interventions. Since the national government relaxed the One Child Policy in 2016, the Family Planning Commission, now part of NHC, has shifted its focus from managing the population quantity to improving the quality of human capital, including improving investments in ECD (Wu, Young, & Cai, 2012).

We find that the center-based parenting intervention did not significantly improve the development outcomes (skills) of children in the program. The intervention did, however, produce increases in caregiver investments in children, including increased material investments in toys and picture books, increased time investments in stimulating parenting activities, and improved parenting skills.

The center-based parenting intervention was significantly less effective than was the home-based intervention, producing significantly smaller average impacts on child skill development and caregiver investments. Further analysis of the two interventions indicates that this difference may be due to the differing nature of compliance, or participation, in the two interventions. Whereas parents of children with low baseline cognitive skills tended to select out of the center-based parenting program (i.e., their levels of participation were lower than were the parents of children with higher baseline cognitive skills), the home-visiting parenting program effectively provided parental training to children with both low and high baseline cognitive skills. Because the two programs had larger impacts on children with lower levels of skills before intervention, as has been found with parenting programs in other countries, our findings suggest that the greater compliance of more vulnerable children in the home-visiting program may have led to larger average impacts than in the center-based parenting program.

This study contributes to the growing literature on the effects of parenting interventions delivered through public resources in LMICs. To the best of our knowledge, this study is the first to compare two popular delivery models for parenting interventions, using the same target region, curriculum, and measurements. The results emphasize the importance of program participation in ensuring program effectiveness and cost effectiveness. Due to selection in program compliance, the active home-based delivery model may be more effective and cost-effective than is the more passive form of service delivery through parenting centers in rural areas.

The remainder of this paper is organized as follows. Section 2 presents the sample, experimental design, and empirical approach used to analyze the center-based parenting intervention. Section 3 contains the findings for the center-based parenting intervention. Section 4 provides a comparison of the differences in design and effects between the center-based and home-based parenting interventions, and Section 5 concludes.

## 2 Methods

### 2.1 Sampling and randomization

Our trial of the center-based parenting intervention was conducted in 22 nationally designated poverty counties^1^ in a northwestern province of China. According to statistics from the National Bureau of Statistics of China (NBSC), for 2016, the per capita income of rural residents in the sample province was 9396 yuan ($1337 USD), around the national median of 12,363 yuan ($1759 USD) for China’s rural areas.

Within the sample region, we followed a three-step protocol to select the study sample. First, in each sample county, we obtained a list of all townships from local NHC officials. We excluded the township in each county that housed the county seat (which tend to be wealthier and more urban than the average rural township) as well as townships that did not have any villages with a population of 800 or more. From the remaining townships, we then randomly selected 100 for inclusion in the sample. Second, within each township, we randomly selected one village to participate in the study, totaling 100 villages. To ensure that all sample villages would have the potential space to conduct the center-based parenting intervention, villages that could not supply a 60–80 m^2^ space for the intervention site were excluded. If a village did not have the available space, it was replaced with a randomly selected village from within the same township. Finally, a list of all registered births over the past 24 months was obtained from the local NHC official in each sample village, and all children in the desired age range (6–24 months) and their caregivers were enrolled in the interventional study. In total, 1720 children in 100 villages were sampled at baseline.

**Fig. 1:**
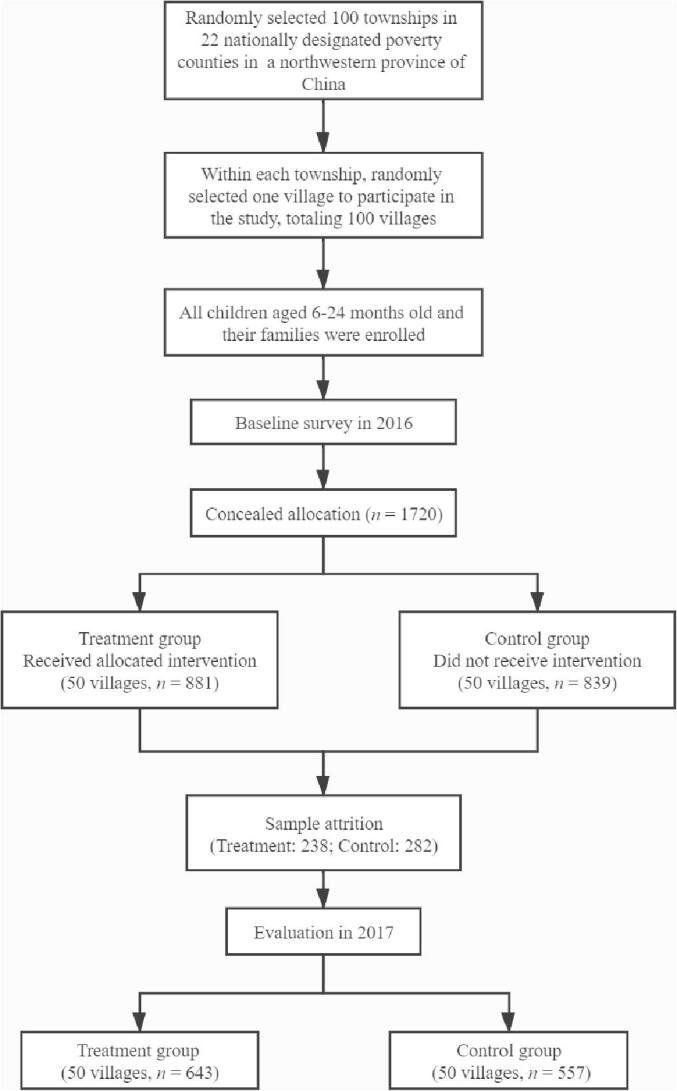
Flowchart of the center-based parenting intervention.

After the baseline survey, the research team randomly allocated 50 sample villages to the treatment arm of the study and 50 villages to the control arm. In total, 881 sample children and their caregivers were assigned to the treatment arm. The remaining 839 children and caregivers were assigned to the control arm (Fig. 1).

### 2.2 Intervention: a center-based parenting program

**Fig. 2:**
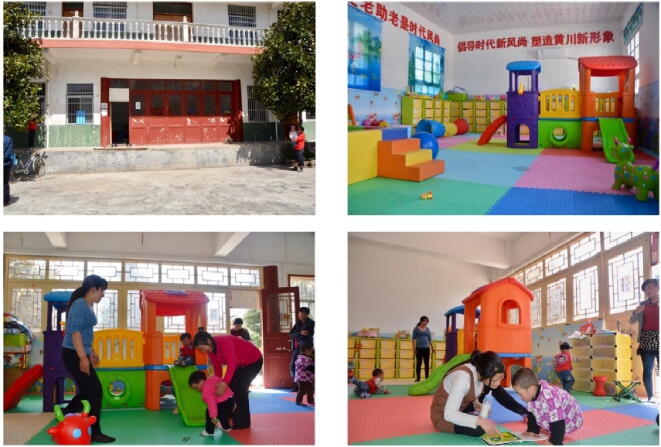
Images of parenting centers established in repurposed buildings in rural villages of China.

In each of the 50 treatment villages, one parenting center was established in a centrally located building (e.g., a repurposed schoolhouse, cultural center, office space) provided by the village committee (Fig. 2). The parenting centers were operated for 12 months, after which we re-assessed ECD outcomes and parental investment. Each parenting center was renovated to be child friendly, with colorful walls, non-lead-based paint, and soft floors. All parenting centers contained a large play area, as well as a standard set of toys, baby books, and decorations provided by the research team. Each parenting center also contained a smaller room for one-on-one parenting training sessions. The parenting centers were designed to be open 5 h a day, 6 days a week. According to monitoring data collected by the research team, the parenting centers were open for an average of 279 days during the first year of operation. Caregivers were encouraged to bring their children to the parenting centers during open hours but were not allowed to leave their children alone in the parenting centers.

*Clockwise from left:* public village building used for parenting center in a sample village; established parenting center in a sample village; caregiver and child reading in a parenting center; children and caregivers playing in parenting center.

Each parenting center also contained a smaller room for one-on-one parenting training sessions. Caregivers were invited to attend weekly parenting training lessons designed to teach interactive parenting practices to stimulate child development. The weekly lessons were about 1 h each; after one year of intervention, caregivers in the treatment group had received about 26 h of one-on-one parental training. In addition to the one-on-one parenting lessons, caregivers were encouraged to bring their children to the centers frequently to engage in free play, socialize with other children, and participate in organized activities such as story time and singing. Caregivers were also invited to bring home toys and books from the parenting centers.

Each parenting center was staffed by two parenting trainers from the local branch of the NHC, who conducted the one-on-one lessons. Before the start of the intervention, all parenting trainers underwent comprehensive training in child development and the structured week-by-week parenting curriculum used in this intervention. The parenting curriculum is adapted from Reach Up and Learn, a curriculum developed and evaluated in Jamaica by Grantham-McGregor, Powell, Walker, and Himes (1991) and used in ECD intervention studies in multiple LMICs (Grantham-McGregor & Smith, 2016). The curriculum aims to teach caregivers how to interact with their children through age-appropriate, stage-based stimulating activities. It consists of weekly interactive training sessions that target caregivers of children aged 6 to 36 months, and each session consists of two age-appropriate activities that involve both caregivers and children. The curriculum was adapted by child development experts in China to fit the context of rural China, and it has previously undergone field testing and evaluation in rural China by members of the research team (Sylvia et al., 2018).

In addition to the two parenting trainers, the research team also hired one local center manager for each parenting center. The center manager was responsible for managing all center activities, including leading organized groups activities (such as storytelling and singing) and recording program participation. For each visit to the parenting center, the center manager recorded the caregiver’s name, the date, and the relationship between the caregiver and the child.

All families in the intervention villages with children aged 6–24 months were contacted by phone once the village’s parenting center was ready for operation and invited to a welcome orientation for families. The orientation was led by the center manager, and attended by a member of the research team. Families were told that the program was part of a government pilot, and that the goal was to provide better child enrichment opportunities in rural areas. The orientation also covered pertinent logistics, such as the center’s hours of operation as well as relevant rules (e.g., children must wear a diaper, etc.). The one-on-one parenting training sessions were also introduced, and each family was assigned a time slot for their weekly session. The vast majority of families attended the orientation; for those who did not, the center manager made house calls to communicate the same information. Families also received reminder phone calls from the center manager prior to their weekly one-on-one training appointment.

### 2.3 Data collection

We collected data in two surveys rounds, which we refer to as the baseline and endline survey, respectively. The baseline survey was conducted in August 2016, after which we began the intervention in treatment villages. The endline survey was conducted one year later, in August 2017. Both surveys were identical and collected data on child development outcomes, parental investment and parenting skills, and demographic characteristics of sample children and households.

*Child developmental outcomes.* The primary outcome of interest in this study are measures of child skill development. In each survey round, children were administered the third edition of the Bayley Scales of Infant Development (BSID-III; Bayley, 2006). The BSID-III includes four scales that assess cognitive, language, motor, and social-emotional skills, respectively. The BSID-III has been formally adapted to the Chinese language and environment and used in multiple studies across rural China (Wang et al., 2019).

The BSID-III test was administered by trained enumerators, using a standardized toy kit and a detailed scoring sheet. A child’s scores on the BSID-III are determined by the child’s performance on a series of tasks, adjusted for age in months and premature birth. The caregiver of each child was present but was not allowed to assist the child during the test. All enumerators attended a one-week intensive training course on BSID-III administration, including 2.5 days of experiential learning in the field, before the survey.^2^

To combine the four BSID-III scales (cognitive, language, motor, and social-emotional) into a single index, we follow Cunha and Heckman, 2008, Cunha et al., 2010 to construct a latent factor measure for child skills, using a dedicated measurement system. We estimated the measurement system separately at baseline and endline as:

(1) $$m_{\mathrm{ik}}^{h}=\mu_{k}^{h}+\alpha_{k}^{h}h_{i}+\delta_{\mathrm{ik}}^{h}$$

where $m_{\mathrm{ik}}^{h}$ is the *k*-th measure of child *i*’s skills, $\mu_{k}^{h}$ is the mean of the *k*-th measure of child skills, $\alpha_{k}^{h}$ is the factor loading of the *k*-th measure, and $\delta_{\mathrm{ik}}^{h}$ is mean zero measurement error term, which is assumed independent of the latent factor, $h_{i}$. This measurement system is assumed invariant to the treatment assignment. That is, any differences in the observed measures of child skills between the control group and treatment group result only from a change in the latent child skill factor. After estimating the measurement system, we use the Bartlett (1937) scoring method to predict the factor score of latent factors for each child, based on the estimated means and factor loadings. The predicted child skill factor is standardized by the distribution of the control group. Further details about the measurement system are described in Appendix A.

*Parental investments and parenting skills.* The parenting curriculum was designed to affect child development by increasing the parental investments and parenting skills of caregivers. To assess the program effects on these secondary outcomes, we collected data on the material investments and time investments as well as parenting skills of the child’s primary caregiver (defined as the individual most responsible for child’s daily care, typically either the mother or paternal grandmother). The primary caregiver was administered a detailed questionnaire adapted from the Family Care Indicators (FCI), which was developed by UNICEF to measure the home environment of young children in developing countries (Frongillo, Sywulka, & Kariger, 2003). Previous studies have demonstrated that the FCI is a reliable measure of parenting and the home environment in developing settings (Hamadani et al., 2010). The FCI has been adapted to the Chinese language and context and used in previous studies across rural China (Wang, Luo, Yue, Tang, & Shi, 2020). A full list of items included in the FCI are reported in Table B1 of Appendix B. As seen in the table, the Cronbach’s alpha coefficient for all items is larger than 0.8, indicating that it has high internal consistency in our sample (Nunnally, 1978).

Using caregiver responses to the FCI, we created two measures of parental outcomes: material investments and time investments. We use six variables to measure caregiver material investments, including sources of play materials, varieties of play materials, total number of play materials, number of picture books, number of books for adults, and number of magazines and newspapers in the home. Time investments were calculated based on whether caregivers had participated in each of five at-home play activities with their child in the past three days: reading books or looking at picture books, telling stories, singing songs, playing with toys, and spending time in naming things, counting, or drawing.

We also collected information on the parenting skills of caregivers. To measure parenting skills, we asked each child’s primary caregiver a series of questions about his or her beliefs and attitudes toward parenting, including whether the caregiver feels a duty to help the baby understand the world, whether the caregiver thinks it is important to play or read with the baby, and whether the caregiver knows how to play or read with the baby.

As we did for child skills, we developed a dedicated measurement system that related all observed measures of caregiver material investments, time investments, and parenting skills to their corresponding latent factors, using Eq. (1). We estimated the measurement system at baseline and at endline for caregiver material investments, time investments, and parenting skills. The predicted material investment factor, time investment factor, and parenting skill factor are all standardized by the distribution of the control group.

*Demographic characteristics.* Finally, we collected demographic information on child and household characteristics. Child characteristics include the child’s gender, age in months, whether the child had a low birth weight, whether the child was born through a natural birth (as opposed to caesarean section), and whether the child was premature. The child’s age and premature birth status were taken from his or her birth certificate. Household characteristics include whether the mother is the primary caregiver, the primary caregiver’s age and level of education, whether the child has older siblings, and whether the household receives social security support through China’s minimum living standard guarantee program.

*Program participation.* In addition to the baseline and endline surveys, we collected information on program compliance for all children and caregivers in the treatment group throughout the one-year intervention. As noted above, the manager of each parenting center recorded each visit to the center, including the child’s name, the date, and the relationship between the caregiver and the child, using a registration form designed by the research team. The research team also made phone calls to randomly chosen households to double-check the accuracy of the records. Based on these records, we calculated the average number of center visits per month for each treatment household.

### 2.4 Baseline characteristics, balance, and attrition of center-based sample

Table 1 presents the descriptive statistics and balance test of baseline characteristics between the control and treatment groups. All *p*-values account for clustering within villages, and the differences in child and household characteristics are all insignificant across the two groups. We also ran a joint significance test for balance by regressing the treatment status on all baseline characteristics reported in the table and tested that the coefficients of all covariates are jointly zero. The *p*-value of this test is 0.898.

**Table 1:** Descriptive statistics and balance test of demographic characteristics.

Variable	Control (*n* = 839)	Treatment (*n* = 881)	*p*-value
*Panel A: Child Characteristics*			
Male (yes = 1)	0.52 (0.02)	0.51 (0.02)	0.562
Age in months	14.49 (0.22)	14.24 (0.21)	0.198
Low birth weight (yes = 1)	0.05 (0.01)	0.04 (0.01)	0.547
Natural birth (yes = 1)	0.65 (0.02)	0.63 (0.02)	0.539
Premature (yes = 1)	0.05 (0.01)	0.04 (0.01)	0.738

*Panel B: Household Characteristics*			
Caregiver age (years)	35.37 (0.62)	35.51 (0.46)	0.794
Caregiver years of schooling	8.18 (0.13)	8.11 (0.19)	0.966
Mother is primary caregiver (yes = 1)	0.70 (0.02)	0.70 (0.02)	0.708
Child has elder siblings (yes = 1)	0.51 (0.02)	0.51 (0.02)	0.999
Household receives social security support (yes = 1)	0.10 (0.01)	0.12 (0.02)	0.599

*Note.* Standard errors presented in parentheses. The *p*-values account for clustering at the village level. An omnibus balance test, conducted by regressing treatment status on all listed covariates and conducting an *F*-test, which cannot reject that the coefficients are jointly zero, yields a *p*-value of 0.898.

Panel A provides the baseline statistics for child characteristics. In our sample, children were just over 14 months old, on average, at baseline. Slightly over half (51%) of the children were male. About 4% of the children were born with low birth weight, and 64% of children were born naturally (the balance, 36%, were born by caesarean section). <5% of sample children were premature at birth.

Panel B provides baseline statistics for caregivers and households. The mother was the primary caregiver in 70% of households.^3^ The average age of primary caregivers (mothers and others) is around 35 years at baseline, and caregivers had slightly over 8 years of schooling on average. Nearly 50% of sample children had older siblings, and approximately 11% of households were receiving social security support at the time of the baseline survey.

Table 2 presents the descriptive statistics and balance tests for measures of child skills at baseline of the center-based parenting program. In our sample, at baseline, the mean scores of the cognitive, language, motor, and social-emotional scales were 96.15, 92.72, 97.42, and 85.92, respectively. For cognitive, language, and social-emotional development, the mean scores in our sample are about 1 *SD* lower than the expected means of healthy population.^4^ In addition, at baseline, 53% of children exhibited cognitive delay, 60% exhibited language delay, 36% exhibited motor delay, and 43% exhibited social-emotional delay. The differences in child development outcomes are all insignificant across the two groups. The *p*-value of the joint significance test is 0.706, which means that all coefficients are jointly zero.

**Table 2:** Descriptive statistics for child skills at baseline.

Variable	Control (*n* = 839)	Treatment (*n* = 881)	*p*-value
*Panel A: BSID-III score*			
Cognitive score	96.07 (0.73)	96.22 (0.83)	0.442
Language score	93.08 (0.75)	92.37 (0.79)	0.503
Motor score	97.90 (0.93)	96.95 (0.99)	0.447
Social-emotional score	85.99 (1.02)	85.84 (0.82)	0.994
*Panel B: Developmental delay*			
Cognitive delay (score < 95.4)	0.53 (0.03)	0.54 (0.03)	0.816
Language delay (score < 96.7)	0.59 (0.02)	0.60 (0.03)	0.913
Motor delay (score < 93)	0.35 (0.02)	0.38 (0.02)	0.337
Social-emotional delay (score < 85)	0.43 (0.03)	0.43 (0.03)	0.982

*Note.* The statistics are the sample mean, and the standard error is presented in parentheses. We regressed the treatment status on all baseline child skill scores and skill delays reported. The *p*-value on each coefficient accounts for clustering at the village level. We conducted an *F*-test, which cannot reject that all coefficients are jointly zero, for which the *p*-value is 0.706.

Table 3 presents the descriptive statistics and balance tests for measures of caregiver material investments, time investments, and parenting skills at the baseline of the center-based parenting program. At baseline, each household had a mean of 2.26 books, and only around 20% of caregivers reported reading books to their child. Only 17% of caregivers reported telling stories to their child, and 41% of caregivers reported singing songs to their child. Only two out of the 16 measures of parental investments and skills were unbalanced (number of magazines and newspapers in the home and whether caregiver knows how to play with the baby), with slightly higher scores in the control group at baseline. The *p*-value of the joint significance test is 0.281, which cannot reject that the coefficients of all baseline measures are jointly zero.

**Table 3:** Descriptive statistics for material investments, time investments, and parenting skills at baseline.

Variable	Control (*n* = 839)	Treatment (*n* = 881)	*p*-value
*Material investments*			
Number of play material sources	2.25 (0.07)	2.31 (0.06)	0.327
Number of play material varieties	3.63 (0.14)	3.68 (0.12)	0.900
Number of picture books	1.68 (0.06)	1.69 (0.05)	0.690
Number of play materials	3.57 (0.09)	3.59 (0.08)	0.278
Number of books (except picture books)	2.29 (0.08)	2.27 (0.07)	0.913
Number of magazines and newspapers	1.62 (0.06)	1.50 (0.05)	0.056*

*Time investments (proportion)*			
Read books or looked at picture books with child in last 3 days	0.19 (0.02)	0.20 (0.02)	0.379
Told stories to child in last 3 days	0.18 (0.02)	0.17 (0.02)	0.933
Sang songs with child in last 3 days	0.42 (0.03)	0.41 (0.02)	0.617
Played with the child with toys in last 3 days	0.67 (0.03)	0.69 (0.02)	0.321
Spent time with child in naming things, counting, or drawing in last 3 days	0.41 (0.03)	0.42 (0.02)	0.978

*Parenting skills (1–7 likert scale)*			
Caregiver feels duty to help baby understand the world	6.29 (0.12)	6.13 (0.11)	0.134
Caregiver finds it important to play with baby	5.08 (0.11)	5.00 (0.10)	0.843
Caregiver knows how to play with baby	4.83 (0.11)	4.55 (0.10)	0.036**
Caregiver finds it important to read stories to baby	4.33 (0.10)	4.36 (0.09)	0.152
Caregiver knows how to read stories to baby	4.20 (0.11)	3.94 (0.12)	0.105

*Note.* The statistics are the sample mean, and the standard error is presented in parentheses. We regressed the treatment status on all baseline material investments, time investments, and parenting skills. The *p*-value for each coefficient accounts for clustering at the village level. We conducted an *F*-test, which cannot reject that all coefficients are jointly zero, for which the *p*-value is 0.281.

*p < 0.10, **p < 0.05, ***p < 0.01

Attrition was relatively high in both the treatment and control groups in our sample. As shown in Fig. 1, of the 881 children in the treatment group at baseline, only 643 children participated the endline survey, a 27% attrition rate. In the control group, 557 of the original 839 children participated in the endline survey, an attrition rate of 33%. Most of the attrition in our sample is due to family out-migration to other parts of the prefecture, province, or nation. Importantly, as shown in Table B2, the attrition rate is balanced between the treatment and control groups. In Columns 3 and 4, the *p*-value of the Chow test is 0.36, which cannot reject that the correlates of attrition are similar in the control and treatment groups. However, even though we fail to find significant differences, the power of these tests is low given the sample size. As a further robustness check, we present bounding analyses following Lee, 2009, Kling et al., 2007 for effects on child skills and investment outcomes in Table B3 in the appendix. This analysis suggests that the the main findings for the center intervention are robust to attrition.

### 2.5 Estimation strategy

*Average treatment effects.* In a randomized controlled trial, comparisons of the mean(s) of the outcome variable(s) between the treatment control groups provide unbiased estimates of the program effects on outcomes due to random treatment assignment. In this study, following the methods of Bruhn and McKenzie, 2009, Sylvia et al., 2018, we controlled for randomization strata (county) and the baseline value of the dependent variable to increase power. We estimated the intent-to-treat (ITT) effects of the parenting intervention by ordinary least squares (OLS) using the following ANCOVA specification:

(2) $$h_{\mathrm{ijt}}=\alpha_{1}+\beta_{1}D_{\mathrm{ij}}+\gamma_{1}h_{\mathrm{ij}(t-1)}+\tau_{s}+\xi_{\mathrm{ij}}$$

where $h_{\mathrm{ijt}}$ is the skills of child *I* in village *j* at endline, $D_{\mathrm{ij}}$ is a dummy that indicates the treatment assignment of child *i* in village *j*, $h_{\mathrm{ij}(t-1)}$ is the skills of child *i* in village *j* at baseline, and $\tau_{s}$ is the strata (county) fixed effects. The coefficient $\beta_{1}$ captures the ITT effects of the center-based parenting intervention on child skill development. We adjusted robust standard errors for clustering at the village level.

To explore the mechanisms through which the parenting intervention may have affected children’s skill development, we considered the general human capital production function of Cunha et al., 2006, Cunha and Heckman, 2007:

(3) $$h_{t+1}=f_{t+1}\left(h_{t},M_{t+1},T_{t+1},P_{t+1},X_{t},\eta_{t+1}\right)$$

where $h_{t+1}$ and $h_{t}$ are child skills at endline and at baseline, respectively; $M_{t+1}$, $T_{t+1}$, and $P_{t+1}$ are caregiver material investments, time investments, and parenting skills during the intervention period, respectively; $X_{t}$ is baseline household characteristics; and $\eta_{t+1}$ represents random shocks to child skills development.

This production function indicates three possible channels^5^ through which the parenting intervention might affect the child’s skills. These channels are (1) changes in material investments, (2) changes in time investments, and (3) changes in parenting skills. Hence, we estimated the ITT effects of the parenting intervention on these three channels using the same specification as that for child skills (Eq. (2)).

## 3 Results

### 3.1 Program effects on child developmental outcomes

Table 4 presents the estimated ITT effects of the center-based parenting program on child skills. After adjusting for multiple hypothesis we do not find evidence that the 12-month center-based parenting intervention increased child skills in any of the domains examined. The estimated treatment effects on each domain, as well as the composite latent skill factor that combines the four domains are not statistically significant.

**Table 4:** Intention-to-treat (ITT) effects on child’s skills.

Skill	Point Estimate	Standard Error	*p*-value	FDR *q*-values
Cognitive skill (*n* = 1200)	0.112	0.059	0.061	0.3240
Language skill *n* = 1200)	0.011	0.059	0.850	0.7400
Motor skill (*n* = 1200)	−0.047	0.070	0.506	0.5090
Social-emotional skill (*n* = 1200)	−0.106	0.074	0.155	0.3240
**Total child skill factor (*N* = 1200)**	**0.028**	**0.061**	**0.652**	**–**

*Notes.* Child’s skills are all standardized by the distribution of the control group. Each row corresponds to an independent regression, and all regressions control county fixed effects and corresponding baseline skills. OLS estimates are reported, and robust standard errors, clustered at the village level, are presented in the second column. The final column reports q-values that control the false discovery rate (FDR) following the procedure of Benjamini, Krieger, and Yekutieli (2006).

*q < 0.10, **q < 0.05, ***q < 0.01

### 3.2 Mechanisms: program effects on caregiver material investments, time investments, and parenting skills

Table 5 presents the program’s ITT effects on caregiver material investments, time investments, and parenting skills. As shown in Panel A, the program had a small but positive impact on the material investments of caregivers. Among the specific components of material investment, the program produced the largest increases in the number of play material sources and the number of picture books for children in the treatment households. It had little effect on the total number of play materials. This is most likely because the parenting center allowed households to borrow play materials from the center to bring home, which increased sources of play materials for children, but the absolute number of play materials at home did not change when the play materials were returned to the centers. Such a finding is consistent with a systematic review of 21 parenting interventions in LMICs by Aboud and Yousafzai (2015), who found that, when households receive free play materials as part of an ECD intervention, they are less likely to invest in additional play materials for their children.

**Table 5:** Intention-to-treat (ITT) effects on material investments, time investments, and parenting skills.

ITT Effects	Point Estimate	Std. Error	*p*-value	*FDR q-value*
*Panel A: Parental material investments (n = 1200)*				
Number of play material sources	0.112	0.052	0.033	0.105
Number of play material varieties	0.076	0.049	0.123	0.197
Number of picture books	0.128	0.052	0.016	0.105
Number of play materials	0.027	0.056	0.628	0.458
Number of books (except picture books)	0.045	0.047	0.343	0.328
Number of magazines and newspapers	0.045	0.051	0.380	0.328
**Material investment factor**	0.089*	**0.046**	**0.056**	

*Panel B: Parental time investments (n = 1200)*				
Read or look at picture books with child	0.303***	0.061	0.000	0.001
Tell stories to child	0.214***	0.053	0.000	0.001
Sing songs with child	0.172***	0.063	0.008	0.008
Play with child with toys	0.061	0.062	0.329	0.166
Spend time with child naming things, counting, or drawing	0.047	0.051	0.355	0.166
**Time investment factor**	0.246***	**0.062**	**0.000**	

*Panel C: Parenting skills (n = 1200)*				
Caregiver feels duty to help baby understand the world	0.007	0.047	0.876	0.213
Caregiver finds it important to play with baby	0.158**	0.057	0.007	0.010
Caregiver knows how to play with baby	0.149**	0.058	0.012	0.012
Caregiver finds it important to read stories to baby	0.166***	0.057	0.004	0.009
Caregiver knows how to read stories to baby	0.182***	0.056	0.002	0.009
**Parenting skill factor**	0.220***	**0.055**	**0.000**	

*Note.* Each row corresponds to an independent regression, and all regressions control county fixed effects and corresponding baseline material investments, time investments, or parenting skills measures. All outcomes are standardized by the distribution of the control group. OLS coefficient estimates are reported, with standard errors clustered at the village level.

*p < 0.10, **p < 0.05, ***p < 0.01

As also seen in Table 5, we find that the intervention produced positive effects on caregiver time investment (Panel B) and parenting skills (Panel C). In the treatment households, caregivers more actively engaged in positive parenting activities, such as reading books, telling stories, and singing songs with their children, all of which have been shown to benefit early cognitive development (Wang et al., 2019, Yue et al., 2017). Moreover, the intervention increased the skills and confidence of caregivers to engage in these activities. At endline, caregivers of children in the treatment group reported not only stronger beliefs on the importance of playing and reading with children but also being more knowledgeable as to how to play and read with their children.

### 3.3 Compliance

Next, we consider program compliance. Based on administrative records from the parenting centers, treatment households completed a mean of 6.3 center visits per month during the study, which is somewhat less than two visits per week. More than half (55.62%) of treatment households completed no more than four visits per month, or an average of one visit per week. Less than one quarter (22.70%) completed at least 12 visits per month (three visits per week).

### 3.4 Heterogeneous effects

Table 7 examines the heterogeneous effects on child skills (the total child skill factor) of the center-based parenting program by child age and initial levels of child skills, parental investment, and parenting skills. The intervention had larger effects on younger children (6 to 17 months at baseline) compared to older children (18 to 24 months at baseline), but this difference is insignificant.^6^ We also do not find significant heterogeneity by baseline parental investments or parenting skills. The interaction terms in each of these regressions (columns 3–5) are, however, negative, indicating that the program was likely to have had larger effects on children initially receiving higher levels of parental investment and whose caregivers had better parenting skills.

**Table 7:** Heterogeneous effects of the center-based parenting intervention.

Outcome: Total child skill factor at Endline	(1)	(2)	(3)	(4)	(5)
	Baseline characteristics				
	Age < 18 Months	Low Child Skills	Low Material Investments	Low Time Investments	Low Parenting Skills
Treatment (a)	−0.100	−0.038	0.080	0.028	0.060
	(0.106)	(0.084)	(0.073)	(0.078)	(0.081)
Baseline characteristics	−0.088	−0.011	−0.126**	−0.247***	0.008
	(0.090)	(0.107)	(0.061)	(0.071)	(0.066)
Treatment * baseline characteristics (b)	0.186	0.131	−0.103	−0.016	−0.063
	(0.118)	(0.106)	(0.076)	(0.094)	(0.090)
County FE	Yes	Yes	Yes	Yes	Yes
Baseline cognitive skills	Yes	Yes	Yes	Yes	Yes
Treatment effect on those with baseline characteristics (a + b)	0.086	0.093	−0.024	0.013	−0.003
	(0.067)	(0.076)	(0.071)	(0.075)	(0.068)
Observations	1200	1200	1200	1200	1200

*Note.* OLS estimates are reported, and robust standard errors, clustered at the village level, are presented in parentheses. Low child skills are 1 if the child’s baseline total skill factor is less than the median in the sample; Low investments and parenting skills are defined analogously using the respective indices. *p < 0.10, **p < 0.05, ***p < 0.01

## 4 Comparison between the center-based and home-based parenting programs

The center-based parenting program was implemented by the same research team in the same target area as a home-based parenting program evaluated by Sylvia et al. (2018). The evaluations of the two interventions were similar in a number of important ways, which, while absent a head-to-head comparison as part of the same trial, allows us to compare effectiveness and narrow the scope of possible reasons for any difference in average treatment effects. We first compare the two parenting interventions and evaluations in terms of key design features. We then compare treatment effects on primary child development and parental investment outcomes. Finally, we explore four possible reasons for the difference in average treatment effects.

### 4.1 Comparison of evaluation designs

The home-based parenting trial took place from November 2014 to April 2015. Parenting trainers made weekly home visits to families with children aged 18–30 months at the start of the study. A total of 508 children were sampled at baseline. During the home visits, parent trainers were trained to introduce caregivers to set activities and assist caregivers to engage in the activities with their child.^7^ At the end of each weekly session, the materials used for that week’s activities (toys and books) were left in the household to be returned at the next visit. The intervention lasted for six months. The study team found that the program significantly increased infant skill development and that increased investments by caregivers alongside improvements in parenting skills were a major mechanism through which this occurred. Children who lagged behind in their cognitive development and received little parental investment at the onset of the intervention benefited most from the program.

Evaluations of the two parenting interventions were similar in a number of important ways, which, while absent a head-to-head comparison as part of the same trial, allows us to narrow the scope of possible reasons for the difference in average treatment effects. First, both interventions worked with the local NHC officials to recruit trainers in the same way. Second, both interventions used the same age-appropriate, stage-based curriculum. Third, in both interventions, toys, books and other materials were offered to caregivers to bring home. Fourth, the total time of one-on-one instruction with parenting trainers was similar (~26 h total). Fifth, both interventions were implemented in the same region and followed a similar sample selection procedure. Finally, the interventions were evaluated by the same research team, using similar instruments and approaches.

Despite these similarities in the evaluation designs, there are are four main differences that could be large enough to drive differences in impacts: (1) the population of children and caregivers involved in each study; (2) program duration; (3) aspects of outcome measurement; and (4) method through which the interventions were delivered, specifically, through home-based or center-based parenting sessions.

### 4.2 Comparison of average treatment effects

The estimated average treatment effect of the center-based parenting intervention on total child skills and on material and time investments by caregivers was significantly smaller than that of a previous home-visiting intervention evaluated in Sylvia et al. (2018). Table 8 shows raw comparisons across the outcomes examined in both trials. The total skill factor (aggregating Cognitive, Motor, and Socio-emotional skills) is 0.23 *SD*s smaller (*p*-value 0.023) in the center-based parenting intervention (row 1).^8^ The point estimate for the effect of centers on cognitive skill is less than half that of the home visiting parenting program (though statistically insignificant). Similarly, the average treatment effects of the center-based parenting intervention on material investments (0.09 *SD*) and time investments (0.25 *SD*) were significantly smaller than were the effects of the home-visiting intervention on parental investment (0.85 *SD*).

**Table 8:** Differences in estimated treatment effects of parenting centers and home visiting.

Outcome	(1)	(2)	(3)	(4)	(5)
	PC Effect	HV Effect	Difference (PC - HV)	SE of Difference	P-value
Total child skill factor	0.028 (0.061)	0.259*** (0.081)	−0.231	0.101	0.023
Cognitive skill	0.112* (0.059)	0.292^**^ (0.119)	−0.180	0.133	0.176
Motor skill	−0.047 (0.070)	−0.024 (0.120)	−0.023	0.139	0.868
Social-emotional skill	−0.106 (0.074)	−0.010 (0.135)	−0.096	0.154	0.533
Material investment factor	0.089* (0.046)	0.825*** (0.107)	−0.736	0.116	<0.001
Time investment factor	0.246*** (0.062)	0.825*** (0.107)	−0.579	0.124	<0.001
Parenting skill factor	0.220*** (0.055)	0.323^***^ (0.091)	−0.103	0.106	0.331

*Note.* PC denotes the parenting center program, and HV denotes the home visit program. The parental investment measure for the HV trial combines material and time investments. Column 5 reports p-values from independent sample t-tests. The sample size for the parenting center sample is 1,200 and is 503 for the home-visiting intervention.

*p < 0.10, **p < 0.05, ***p < 0.01

### 4.3 Differences in the samples

Although they were conducted in the same region, the inclusion criteria for villages in the two programs differed in that only villages with available space were included in the sample for the parenting center trial. As a result, approximately 15% of villages from the sampling frame were excluded. Despite this difference, however, as shown in Appendix C, the baseline characteristics of the two samples are similar in terms of the static characteristics of children and caregivers. In both programs, approximately half of the sample children were male (49% in home-based program vs. 52% in center-based program, *p*-value = 0.737); few had low birthweight (4%, *p*-value = 0.956); the majority were natural births (69% vs. 64%, *p*-value = 0.797); and the educational level of caregivers was similar (8.55 years vs. 8.15 years, *p*-value = 0.347). Although the share of households that received social security was higher in the home-based program (27% vs. 11%, *p*-value < 0.001), this difference is almost certainly due to the rise of China’s poverty alleviation program, which was beginning to replace part of the social security program in the years that the center-based parenting program was implemented (which was two years after the implementation of the home-based program).

The primary difference between the samples is the mean age of the children. Children in the center-based parenting intervention were 6–24 months old at baseline, whereas children in the home-based parenting intervention were 18–30 months at baseline. By design, children in the home-based intervention were, on average, 10 months older when they were enrolled at baseline (24.45 months vs. 14.36 months, *p*-value < 0.001). This most likely also drives the five-percentage-point difference in the probability that the mother was the primary caregiver at the start of the intervention, as many mothers out-migrate to work in urban areas when their children grow into toddlerhood (Yue, Bai, Shi, Luo, Rozelle, Medina, & Sylvia, 2020).

The difference in the age profiles of children in the center-based and home-based parenting programs, however, appears unlikely to be the primary source of difference in the average impacts that we observe. The center-based parenting program enrolled children aged 6–24 months at baseline; although children age 6–17 months at baseline were younger than the children in the home-based program (who were 18–30 months at baseline), the children in the center-based program who were 18–24 months were of similar age to those in the home-based program. As shown in Table 7, Column 1, in the case of the center-based parenting program, the intervention had larger (and significant) effects on younger children (6–17 months at baseline) than on older children (18–24 months at baseline). There were no detectable effects on the older cohort, which corresponds to the age of the children in the home-visit evaluation, and the difference in average effects among this age group in the center- and home-based interventions is larger than the difference observed in the full samples of the two studies. This difference in age profiles across the two studies, therefore, cannot be what is driving *smaller* average impacts for the center-based parenting program.

### 4.4 Differences in outcome measurement

Although the constructs, or domains, of child development that were used as primary outcomes in both studies were these same, there were some differences in how these were measured. The center-based parenting trial measured child skills at endline using the BSID-III. Version 3, however, was only recently adapted for use in China and had not been available during the home visiting intervention. The evaluation of the home-based parenting intervention used Version 1 of the Bayley Scales of Infant Development (BSID-I) in the younger half of the sample (children below 30 months) and the Griffith Mental Development Scales (GMDS) for children above 30 months at endline.

Focusing on cognitive skills, where we find evidence of positive impacts of the center-based parenting intervention, our results suggest that this difference also is not a significant driver of the difference in average outcomes. First, the home-visiting parenting program had a significant positive effect of 0.26 *SD* on the BSID-I Mental Development Index (MDI) among children who were administered the BSID-I at endline, which is more directly comparable to the BSID-III, used to evaluate the impact of the center intervention, than is the GMDS. The main difference is that, although the BSID-I MDI was designed to measure both cognitive and language development, these two constructs were measured separately (as a cognitive skill index and language skill index) in the BSID-III. That we find no effect on the language skill index in the center-based parenting intervention, but see effects on the cognitive skill index, suggests that, if the BSID-I were to have been used to evaluate the center-based parenting intervention, effects on the MDI would have been smaller than the effects that we find on the BSID-III cognitive skill index, assuming that the splitting of cognitive and language items is the main difference in the measures and depending on the degree to which language items are weighted in the MDI.

### 4.5 Differences in program duration

Having ruled out differences in the sample populations enrolled in each study and in measurement of outcomes, we turn to differences in features of the interventions as possible sources of the different impacts. A first major difference between the two programs was the duration of the intervention. The center-based parenting program lasted for 12 months, while the home-based intervention lasted only 6. This means that children were exposed not only for a longer time in the center-based evaluation but also to more of the curriculum content in the one-on-one sessions. The older age group that was common to both programs was exposed to the final year of the curriculum in the center-based parenting intervention but only to the final six months in the home-based intervention. All else equal, for this to be the reason for larger effects in the home-based parenting intervention, it would need to be the case that children in the center-based parenting intervention benefited less (in absolute terms) than did children in the home-based intervention from the last six months of the curriculum because they had already participated for six months. This scenario is unlikely, particularly given evidence from other contexts of dynamic complementarities or that there is a higher marginal return to human capital from a given investment for children with a larger stock of initial skills (Aizer & Cunha, 2012).

### 4.6 Differences in delivery method

A final major difference between the interventions is, of course, how they were delivered. In the center-based parenting intervention, parenting trainings were delivered in a centralized location in the village to which caregivers had to bring their children, which can be considered a more *passive* delivery method, as it relies on caregivers’ choosing to bring children to the centers. In contrast, the home-based parenting intervention was delivered directly to caregivers in their own homes, a more *active* method of delivery, as caregivers did not have to choose to travel to the training.^9^

The most plausible way through which this difference in delivery models could affect program effectiveness is through uptake or compliance (i.e., visits to the parenting center or in-home sessions with a parenting trainer). That the center-based parenting intervention was delivered in a passive manner, as opposed to the more active delivery of home visits, raises participation costs on households and creates more scope for selection into and out of program participation. As a result, average effects could differ between the two programs due to how individuals selected into participation and how that affected who was treated by the intervention. We explore this possibility by first comparing the patterns of participation between the interventions and then by examining how this pattern maps onto intervention effects.

Differences in compliance could operate in terms of both the overall average level of compliance and the composition of those participating in each intervention. In terms of average participation, treatment households in the center intervention completed a mean of 6.3 center visits per month during the study, more than participants in the home-visiting intervention would have received under full compliance (1 visit per week). For one-to-one sessions, average participation in the center-based parenting intervention was similar to that of the home-based parenting intervention.

The composition of those taking up the intervention, however, differed substantially between the two programs. Table 9 presents the correlation between participation and baseline cognitive skills/parenting outcomes in the center-based and home-based parenting programs. In the center-based program, caregivers of children with low baseline cognitive skills were less likely to visit the center, while baseline investments and parenting skills were not significantly correlated with compliance. In contrast, in the home-based program, children with lower baseline investments completed a greater number of sessions, while baseline cognitive skills and parenting skills were not significantly correlated with program compliance. These correlations show that children with low initial cognitive skills tended to select out of the center-based program, whereas the home-visiting program tended to select out children who were already receiving a relatively high level of investment by caregivers.

**Table 9:** Correlation between program compliance and baseline child cognitive skills/parental outcomes in center-based parenting intervention and home-visiting intervention.

Outcome: Average sessions per month	(1)	(2)	(3)	(4)	(5)
	PC	HV	Difference (PC - HV)	SE of Difference	P-value
Cognitive Skills	0.472* (0.278)	−0.013 (0.086)	0.485	0.291	0.096
Material investment	0.090 (0.436)	−0.130* (0.066)	0.220	0.441	0.618
Time investment	0.050 (0.281)	−0.130* (0.066)	0.180	0.289	0.533
Parenting skill	0.128 (0.337)	0.032 (0.080)	0.096	0.346	0.781

*Note.* PC denotes the parenting center program, and HV denotes the home visit program. The parental investment measure for the HV trial combines material and time investments. Coefficients are from regressions controlling for baseline characteristics and county fixed effects. Column 5 reports p-values from independent sample t-tests. The sample size for the parenting center regressions is 792 and is 210 for the home-visiting intervention (treatment groups only).

*p < 0.10, **p < 0.05, ***p < 0.01

Whether this difference in the pattern of compliance affected the average impacts of each program, however, depends on how different children were affected by participation in each intervention. More specifically, it depends on how the technology of skill formation that relates increased investment to skills varies by baseline characteristics. Table 10 presents the heterogeneous treatment effects by baseline characteristics in the home-based intervention and compares these to the results presented for the center-based parenting intervention in Table 7. Children with low baseline skills improved significantly more than did those with high baseline skills. The estimated effect on low-ability children was 0.4 SD due to the home-visiting program, significant at the 1% level, whereas there was no effect on those with high baseline skills. The impact on children with low initial skill levels is significantly larger in magnitude to what we find for the center-based parenting intervention (Panel B, Column 1).

**Table 10:** Heterogeneous effects of the home-based intervention & comparison to center-based parenting intervention.

Outcome: Total child skill factor at Endline	(1)	(2)	(3)
	Baseline characteristics		
	Low Child Skills	Low Investments	Low Parenting Skills
*Panel A: Heterogeneous effects of the home-based intervention (n = 503)*			
Treatment (a)	0.067	−0.006	0.238**
	(0.099)	(0.101)	(0.107)
Baseline characteristics	0.009	−0.145	0.107
	(0.176)	(0.122)	(0.100)
Treatment * baseline characteristics (b)	0.331**	0.416**	−0.071
	(0.154)	(0.169)	(0.148)
County FE	Yes	Yes	Yes
Baseline cognitive skills	Yes	Yes	Yes
Treatment effect on those with baseline characteristics (a + b)	0.398***	0.409***	0.167
	(0.121)	(0.128)	(0.110)

*Panel B: Comparison to center-based parenting intervention*			
Treatment effect on those with baseline characteristics (a + b in Table 7)	0.093 (0.076)	0.013 (0.075)	−0.003 (0.068)
Difference (PC-HV)	−0.305**	−0.396***	−0.17
SE of Difference	0.143	0.148	0.129
P-value	0.032	0.007	0.19

*Note.* OLS estimates are reported, and robust standard errors, clustered at the village level, are presented in parentheses. Low child skills are 1 if the child’s baseline total skill factor is less than the median in the sample; Low investments and parenting skills are defined analogously using the respective indices. The first row in Panel B re-presents estimates from Table 7. P-values in Panel B are from independent sample t-tests comparing the subgroup treatment effects in the two interventions. Because material and time are not separated in the investment index from the home-visiting intervention, Column (2) in Panel B compares investments in the home-visiting intervention to time investments in the parenting center intervention. *p < 0.10, **p < 0.05, ***p < 0.01

The pattern of heterogeneous effects also differs significantly in terms of initial investments. The effects by baseline investments are, in fact, opposite in the center-based and home-based parenting programs: The effects of the home-based program on children with low baseline investments are 0.42 SD higher than children with high baseline investments, significant at the 5% level (Table 10, Column 2). This is in contrast to the center-based parenting intervention, which, as discussed, had significantly larger impacts on children with higher levels of initial investment (Table 7, Columns 3 and 4). Among children with low initial investments, the effect of home visiting was almost 0.4 SD higher than the canter-based parenting intervention.

In summary, we find that, in the center-based parenting intervention, caregivers of children with low levels of baseline skills tended to select out of participation, whereas they did not in the home-based parenting intervention. In the home-based intervention, those who selected out tended to be caregivers with higher levels of initial investment. Because children with lower levels of initial skills and whose caregivers are investing less tend to benefit more from parenting interventions, this is a likely cause for lower average impacts of the center-based program.

## 5 Conclusion

Little is known about the relative effectiveness of different delivery models for parenting interventions in LMICs. We evaluated the effects of a free center-based parenting intervention on ECD outcomes and parenting practices, using data from a randomized trial across 100 villages in rural northwestern China. We also compared the effects of the center-based intervention with those of a home-based parenting intervention conducted by the same research team in the same region and using the same parenting curriculum and public service system as the center-based intervention (Sylvia et al, 2018).

We find that the center-based parenting intervention produced positive and significant impacts on caregiver material investments, time investments, and parenting skills. The center-based program did not have significant effects on child outcomes on average and the effects of the center-based intervention on parenting outcomes were significantly lower than those of the home-based intervention. According to our analysis, this is likely due to the selection of families into the programs in terms of compliance: In the center-based program, caregivers of children with low baseline cognitive skills tended to participate at a lower rate, whereas, in the home-based program, caregivers who were already investing in their children at higher levels were the ones who tended to not participate. Our findings suggest that greater compliance by families with more vulnerable children in the home-based program contributed to the larger impacts (on average) than the center-based program. Thus, at least as implemented, it seems clear that the home-based program was more effective in raising the cognitive skills of the children who presumably are the ones who need the most attention. Moreover, given the evidence from other studies that parenting interventions may have greater impacts on cognitive skills when children are younger (Attanasio et al., 2020, Heckman et al., 2020), if the children in the home-based program were the same age as children in the center-based intervention at baseline (6–24 months, instead of 18–30 months), it is possible that we would have seen an even greater difference in program imapcts of the home-based intervention on child cognitive skills.

Are there any actions that can be taken by local policymakers (or program managers) that could help increase compliance in the center-based parenting intervention? In other nations that are running parental support programs, the government has sought to encourage caregivers to attend the training events by offering conditional cash transfers (Fernald, Gertler, & Neufeld, 2008). Conditional cash transfers offer a certain amount of money to participants in exchange for their participation. The underlying logic of the transfers is that there is a social benefit to increased participation and ensuring higher future benefits for society. Unfortunately, with the exception of certain environmental programs (e.g., Grain for Green—Uchida, Rozelle & Xu, 2009), national and local government officials have been unwilling to use CCTs as a policy tool to improve compliance to any program in the areas of education, health or ECD (Zhou, Jiang, & Wang, 2020).

Beyond differences in effectiveness, there are also clear differences in the cost associated with providing parental training to children in the study areas. The costs of the home-based intervention include mainly the salaries of the parenting trainers (and monitors of the trainers) and the supply of toys and books (both originals and replacements). The costs of the center-based intervention included these same costs—the salaries of parenting trainers and the supply of toys and books—and because the number of trainers, toys, and books per village were the same in both the home-based and center-based programs, these elements had nearly identical costs. In the case of the parenting centers, however, there were additional costs, including the salary of the day-to-day center manager and the cost of the utilities of the center, including the cost to turn the center into a child-safe play space. Full cost accounting also would include the rental fee for the center building/space (although in this particular intervention, villages provided it for free). In this way, then, it is obvious that the center-based intervention was more expensive than was the home-based one. Although per-child costs of parenting centers are lower in larger communities with more children, we find evience that the effect of parenting centers on child skills is likely to be smaller in larger villages. We estimate the the effect of parenting centers in large villages in our sample (with more than the median number of children) to be −0.22 standard deviations less than smaller villages (−0.08 SD in large villages vs 0.12 SD in small villages; unadjusted p-value of difference = 0.083). This suggests that, in the case of rural China, home-visiting would be more cost-effective even in villages with more children.

These findings complement two previous studies that compare the relative cost-effectiveness of home- and group-based parenting interventions in other LMIC settings. Walker et al. (2015) compared the benefit-to-cost ratios of a group program in Antigua, Barbuda, St. Lucia, and Jamaica to a home-based one-on-one program in Jamaica, finding that the ratios of long-term benefits over costs range from 5.3 to 9.9 and from 3.8 to 7.1 for the center-based and home-based programs, respectively. Second, Grantham-McGregor et al. (2020) compared short-term cost-effectiveness across home-based (one-on-one) and group parenting training in the context of poor, rural regions in India. finding that home-based and center-based parenting training were equally effective, but the home-based intervention was 3.5 times more costly than center-based group service delivery (with annual costs of USD 135 and USD 38, respectively). As with the center-based parenting intervention in the current study, group interventions in these studies took placed in centralized venues. A primary difference, however, was that in the intervention presented here parenting sessions were still one-on-one and parenting centers were equipped with toys and books and were available for use 6 days a week and 6 h per day. Installing the dedicated parenting centers became more costly than using publicly available spaces (as did the group interventions in these previous studies), although it could have also provided greater benefits in facilitating larger and more persistent impacts through better monitoring of service quality, more efficient allocation of toys and books, and improved peer interaction for children and caregivers, when they visited the centers on a more regular basis.

This is the first study to compare two viable delivery models for parenting interventions, using the same research team, program, and methods. The results provide insight into the effects of parenting interventions delivered through existing public resources in developing countries. Our findings also shed light on the underlying mechanisms for the differences in intervention effects, that is, differences in the patterns of compliance between the two delivery models.

We also acknowledge two main limitations to this study. First, because the experiment was conducted in only one underdeveloped rural area in Western China, the results may not be generalizable to all LMICs. In addition, this study compared only the short-term treatment effects of the center-based and home-based parenting programs. Although we find the home visiting program was effective in the short-term, a number of studies have shown patterns of large increases in skills at the conclusion of interventions, followed by rapid fade-out (Bailey et al., 2017, Barnett, 2011). In low- and middle-income countries, three recent studies for instance have found different results in medium-run follow-ups. A follow-up study of a parenting intervention in Pakistan finds that initial improvements in early skills of two-year-old infants persist two years after program completion (Yousafzai et al., 2016). In contrast, a follow-up study of a similar integrated ECD intervention in Colombia finds that initial gains in early skills faded out two years after that program (Andrew et al., 2018). Similarly, Özler et al. (2018) find an impact of an integrated parenting intervention on language and socio-emotional development of children in Malawi at 18 months but not at a 36-month follow-up. Future research follow up on the children in both the center-based and home visiting parenting interventions to investigate differences in treatment effects of the two programs over time.

Despite these limitations, the results have important implications. Our findings suggest that how program design affects participation is critical to overall program effectiveness. Due to selection of participation in both programs, the home-based delivery model may be more effective, and cost effective, than is the center-based delivery model. Alternatively, increasing program participation of vulnerable children and their caregivers, for example through conditional cash transfers or other measures, would substantially increase the benefits of the center-based intervention, which could play an active role in shaping early cognitive skills in poor rural areas.^10^ These results may have implications for other social services, suggesting that more passive delivery models (as with parenting centers) may select out participants most likely to benefit compared to more active forms of delivery (as with home visits). Designing interventions to encourage participation among those most likely to benefit may improve the effectiveness of these programs.

## Footnotes

1 To focus effort on its rural poverty alleviation program, the central government in China used per capita net income to designate certain rural counties as national poverty counties (The State Council of the People’s Republic of China, 2006).

2 All enumerators had previous experience administering the BSID as part of previous projects.

3 In rural areas, it is common that caregivers for children are grandmothers while their mothers out-migrate to urban areas for work (Yue et al., 2020).

4 The BSID-III was designed to have a mean score of 100 with a SD of 15 for a healthy population (Bayley, 2006). However, empirical evidence from reference populations around the world found higher means and lower SDs. As a result, when using the traditional cut-off score of 85 (i.e., 1 SD below the mean), the literature has shown that the prevalence of moderate developmental delay was underestimated (see, Anderson et al., 2010; Lowe et al., 2012). According the empirical literature, researchers have shown that, in a healthy reference population, the mean BSID-III score (SD) is expected to be 105 (9.6), 109 (12.3), 107 (14), and 100 (15) for the cognitive scale, language scale, motor scale, and social-emotional scale, respectively (Lowe et al., 2012; Serenius, 2013; Bayley, 2006; Bos, 2013). These cut-offs have been used in published research by Zhang et al. (2018), Wang et al. (2019), and Luo et al. (2019).

5 Another potential channel is that the intervention could change production technology. In a study of an ECD intervention in Colombia, however, Attanasio et al. (2014) do not find support for this channel. We therefore assume this channel to be negligible and focus on the reduced-form impacts on inputs to the production function.

6 We split the sample at 18 months of age to facilitate comparison with the home-visiting program (Section 4).

7 The workload of parenting trainers in each intervention was also comparable. Parenting trainers in the center-based intervention were assigned 8 sessions per week on average while trainers in the home visiting intervention were assigned approximately 5 on average. However the total workload of trainers conducting home visits was slightly higher per session given travel and preparation time.

8 The analysis in this section is exploratory. Accordingly, significance is not adjusted for multiple hypothesis testing.

9 Another possibility is that the center-based delivery method may have encouraged more interaction between children and caregivers in the same village, whereas the home-based intervention did not offer that opportunity. It may be that negative peer effects in the parenting centers offset positive effects of increased investments from the intervention. For example, externalizing behaviors in some children who attended the parenting centers may have negatively affected the cognitive development of their peers. Although some negative peer effects of this type are plausible, however, we believe that it is unlikely that these were large enough to explain much of the difference in average effects compared to the home-based intervention.

10 In the context of parenting programs, evidence from this study and elsewhere show that children with lower skill levels and those receiving less parental investment are most likely to benefit. Although these characteristics would be expensive to measure for the purposes of targeting, previous analyses show that characteristics, such as family wealth and other household characterisitcs, tend to be surprisingly unpredictive of child development levels. One factor that does seem to be consistently predictive of child development is caregiver education, lending to the idea that low parental investment, at least in rural China, is mainly a result of low knowledge of modern parenting practices (Yue et al., 2017).
