# Supplementary material for: Passive versus active service delivery: Comparing the effects of two parenting interventions on early cognitive development in rural China
Source: World Dev. 2022 Jan;149:105686. doi: 10.1016/j.worlddev.2021.105686 (PMC8566276; doi:10.1016/j.worlddev.2021.105686)
Supplement: Supplementary data 1 [file mmc1.docx]

**Appendices to “Passive versus active service delivery: Comparing the effects of two parenting interventions on early cognitive development in rural China”**

**Appendix A. Measurement System**

In this study, we construct latent factor measures for child skills (a composite of the BSID-III cognitive, language, motor and social-emotional subscales) as well as caregiver material investments, time investments, and parenting skills. To preliminarily identify relevant measures of latent factors for the measurement system, we used exploratory factor analysis (EFA). We used Cattell’s (1966) scree plot and Horn’s (1965) parallel analysis, both of which are reported in Table B1. Both analyses show that one latent factor should be extracted from the measures for child skills, material investments, time investments, and parenting skills and at baseline.

**Table A1.** EFA to Determine the number of latent factors

|  | Cattell’s scree plot | Horn’s parallel analysis |
| --- | --- | --- |
| Material investments at baseline | 1 | 1 |
| Time investments at baseline | 1 | 1 |
| Parenting skills at baseline | 1 | 1 |
| Child skills at baseline | 1 | 1 |

Table A2 reports EFA-estimated rotated factor loadings on measures of child skills, material investments, time investments, and parenting skills at baseline. We find that all measures at baseline load strongly on the first factor.

**Table A2.** Estimated rotated factor loadings by EFA on child skills, material investments, time investments, and parenting skills at baseline

| Model | First Factor |
| --- | --- |
| One-factor model of child skills |  |
| Cognitive skills | 0.612 |
| Language skills | 0.614 |
| Motor skills | 0.626 |
| Social-emotional skills | 0.210 |
| One-factor model of material investments |  |
| Number of play material sources | 0.539 |
| Number of play material varieties | 0.775 |
| Number of picture books | 0.601 |
| Number of play materials | 0.714 |
| Number of books (except picture books) | 0.599 |
| Number of magazines and newspapers | 0.517 |
| One-factor model of time investments |  |
| Read books or looked at picture books with child in last 3 days | 0.560 |
| Told stories to child in last 3 days | 0.614 |
| Sang songs with child in last 3 days | 0.586 |
| Played with the child with toys in last 3 days | 0.457 |
| Spent time with child in naming things, counting, or drawing in last 3 days | 0.489 |
| One-factor model of parenting skills |  |
| Caregiver feels duty to help baby understand the world | 0.654 |
| Caregiver finds it important to play with baby | 0.722 |
| Caregiver knows how to play with baby | 0.630 |
| Caregiver finds it important to read stories to baby | 0.643 |
| Caregiver knows how to read stories to baby | 0.564 |

Table A3 shows the measurement system for the child skill factor, and Table B4 reports the measurement system for the latent factors of caregiver material investments, time investments, and parenting skills. The first column of each table contains the factor loadings. We normalized the factor loading of the first measure to equal 1 in both rounds, and, thus, this measure defines the scale of the latent factor. Following Attanasio et al. (2014) and Sylvia et al. (2018), we also calculated the signal-to-noise ratio to measure the percentage of each measure’s variance that is driven by signal, which is reported in the second column. This assesses the amount of information contained in each measure. For example, we calculated this ratio for the *k*-th measure of the parenting skill factor as the following specification:

$$S_{k}^{P}=\frac{\alpha_{k}^{2}Var(P)}{\alpha_{k}^{2}Var\left( P \right)+Var(\delta_{k})}$$

where the *k*-th measure of parenting skill factor is written as the following simplified notation:

$$m_{k}^{P}=\mu_{k}+\alpha_{k}P+\delta_{k}$$

We find that the information contained in different measures can vary significantly, even for the same latent factor, and signal cannot account for 100% of the variance for most measures. This indicates that the latent factor approach is useful to reduce measurement errors in modeling child skills and parenting outcomes.

**Table A3.** Measurement system for child skills

| Latent Factor | Measurement | Factor loading | % Signal |
| --- | --- | --- | --- |
| Child skills at baseline |  |  |  |
|  | Cognitive skills | 1 | 43% |
|  | Language skills | 1.09 | 43% |
|  | Motor skills | 1.38 | 45% |
|  | Social-emotional skills | 0.37 | 4% |
| Child skills at endline |  |  |  |
|  | Cognitive skills | 1 | 51% |
|  | Language skills | 1.24 | 60% |
|  | Motor skills | 1.13 | 40% |
|  | Social-emotional skills | 0.23 | 2% |

**Table A4.** Measurement system for material investments, time investments, and parenting skills

| Latent Factor | Measurement | Factor loading | % Signal |
| --- | --- | --- | --- |
| Material investments: B |  |  |  |
|  | Number of play material sources | 1 | 29% |
|  | Number of play material varieties | 2.72 | 66% |
|  | Number of picture books | 1.15 | 39% |
|  | Number of play materials | 1.24 | 52% |
|  | Number of books (except picture books) | 1.32 | 33% |
|  | Number of magazines and newspapers | 0.89 | 25% |
| Time investments: B |  |  |  |
|  | Read books or looked at picture books with child in last 3 days | 1 | 35% |
|  | Told stories to child in last 3 days | 1.07 | 44% |
|  | Sang songs with child in last 3 days | 1.29 | 37% |
|  | Played with the child with toys in last 3 days | 0.87 | 19% |
|  | Spent time with child in naming things, counting, or drawing in last 3 days | 1.01 | 23% |
| Parenting skills: B |  |  |  |
|  | Caregiver feels duty to help baby understand the world | 1 | 47% |
|  | Caregiver finds it important to play with baby | 1.05 | 58% |
|  | Caregiver knows how to play with baby | 1.07 | 40% |
|  | Caregiver finds it important to read stories to baby | 0.97 | 42% |
|  | Caregiver knows how to read stories to baby | 1.04 | 29% |
| Material investments: E |  |  |  |
|  | Number of play material sources | 1 | 15% |
|  | Number of play material varieties | 4.07 | 79% |
|  | Number of picture books | 2.06 | 43% |
|  | Number of play materials | 0.87 | 28% |
|  | Number of books (except picture books) | 1.41 | 19% |
|  | Number of magazines and newspapers | 0.99 | 14% |
| Time investments: E |  |  |  |
|  | Read books or looked at picture books with child in last 3 days | 1 | 43% |
|  | Told stories to child in last 3 days | 1.07 | 50% |
|  | Sang songs with child in last 3 days | 1.07 | 46% |
|  | Played with the child with toys in last 3 days | 0.81 | 28% |
|  | Spent time with child in naming things, counting, or drawing in last 3 days | 0.69 | 20% |
| Parenting skills: E |  |  |  |
|  | Caregiver feels duty to help baby understand the world | 1 | 16% |
|  | Caregiver finds it important to play with baby | 1.69 | 33% |
|  | Caregiver knows how to play with baby | 2.45 | 39% |
|  | Caregiver finds it important to read stories to baby | 2.12 | 37% |
|  | Caregiver knows how to read stories to baby | 2.86 | 39% |

*Note.* B = Baseline; E = Endline

**Appendix B: Data and Sample in the Center-Based Intervention**

**Table B1.** Family Care Indicators (FCI) Scale

|  | Cronbach’s Alpha | |
| --- | --- | --- |
| Subscale | Baseline | Endline |
| Source of play materials |  |  |
| Homemade toys | 0.81 | 0.81 |
| Household objects | 0.81 | 0.82 |
| Things from outside | 0.81 | 0.82 |
| Toys bought from store | 0.81 | 0.82 |
| Varieties of play materials |  |  |
| Things that make/play music | 0.80 | 0.81 |
| Things for drawing/writing | 0.80 | 0.81 |
| Picture books for children (excluding schoolbooks) | 0.80 | 0.81 |
| Things meant for stacking, constructing, building (blocks) | 0.80 | 0.81 |
| Things for moving around (e.g., balls, bats) | 0.80 | 0.82 |
| Toys for learning shapes and colors. | 0.80 | 0.81 |
| Things for pretending (e.g., dolls, tea set) | 0.81 | 0.82 |
| Play activities |  |  |
| Read books or looked at picture books with child in last 3 days | 0.80 | 0.81 |
| Told stories to child in last 3 days | 0.80 | 0.81 |
| Sang songs with child in last 3 days | 0.80 | 0.81 |
| Played with the child with toys in last 3 days | 0.80 | 0.81 |
| Spent time with child in naming things, counting, or drawing in last 3 days | 0.80 | 0.81 |
| Household books |  |  |
| Number of books for adults in the home | 0.80 | 0.81 |
| Picture books |  |  |
| Number of picture books for children in the home | 0.80 | 0.80 |
| Play materials |  |  |
| Number of play materials in the home | 0.80 | 0.81 |
| Magazines |  |  |
| Number of magazines and newspapers in the home | 0.80 | 0.81 |
| Total | 0.81 | 0.82 |

*Note.* The items in the former three subscales (Source of play materials, Varieties of play materials, and Play activities) are scored as yes = 1 and no = 0 (Presence or absence of play material or activity). The four other items (Household books, Picture books, Play materials, Magazines) are scored in terms of the quantity.

**Table B2.** Analysis of sample attrition

|  | (1) | (2) | (3) | (4) |
| --- | --- | --- | --- | --- |
| Attrition | Full sample | Full sample | Control | Treatment |
| Treatment | -0.062  (0.054) | -0.067  (0.052) |  |  |
| Male |  | 0.038**  (0.019) | 0.027  (0.026) | 0.047  (0.028) |
| Age in months |  | -0.001  (0.002) | 0.001  (0.003) | -0.003  (0.003) |
| Low birth weight |  | 0.049  (0.072) | 0.093  (0.112) | -0.011  (0.080) |
| Natural birth |  | -0.033  (0.024) | -0.053  (0.037) | -0.017  (0.030) |
| Premature |  | -0.042  (0.059) | -0.096  (0.076) | 0.017  (0.086) |
| Caregiver’s age |  | 0.004**  (0.002) | 0.007***  (0.002) | -0.0002  (0.003) |
| Caregiver’s year of schooling |  | 0.008**  (0.004) | 0.011*  (0.007) | 0.006  (0.005) |
| Mother is the primary caregiver |  | 0.078*  (0.043) | 0.129**  (0.051) | 0.002  (0.075) |
| Child who has older siblings |  | -0.081***  (0.025) | -0.115***  (0.037) | -0.043  (0.033) |
| Welfare household |  | 0.036  (0.051) | -0.010  (0.059) | 0.075  (0.078) |

*Note.* OLS estimates are reported in the table; robust standard errors are presented in parentheses, clustered at the village level. The *p*-value of the Chow test in Columns 3 and 4 is 0.36, which cannot reject that the correlates of attrition are similar in the control (Column 3) and treatment groups (Column 4).

**p* < 0.10, ***p* < 0.05, ****p* < 0.01

| **Table B3:** Attrition Analysis for Parenting Center Intervention Effects on Child Skills and Parental Investment | | | | | | | | |
| --- | --- | --- | --- | --- | --- | --- | --- | --- |
|  | Lower Bounds | | | Un-adjusted | IPW | Upper Bounds | | |
| Outcomes | (-).25 SD | (-).1 SD | Lee |  |  | Lee | (+).1 SD | (+).25 SD |
|  | (1) | (2) | (3) | (4) | (5) | (6) | (7) | (8) |
| Cognitive skill (*n* = 1200) | -0.011 | 0.069 | -0.018 | 0.112* | 0.124** | 0.302*** | 0.174*** | 0.254*** |
|  | (0.046) | (0.045) | (0.070) | (0.059) | (0.060) | (0.068) | (0.045) | (0.046) |
| Language skill *n* = 1200) | -0.126*** | -0.048 | -0.170** | 0.011 | 0.007 | 0.140** | 0.056 | 0.134*** |
|  | (0.046) | (0.044) | (0.073) | (0.059) | (0.061) | (0.067) | (0.043) | (0.044) |
| Motor skill (*n* = 1200) | -0.154*** | -0.075 | -0.212*** | -0.047 | -0.040 | 0.198*** | 0.031 | 0.110** |
|  | (0.053) | (0.052) | (0.080) | (0.070) | (0.072) | (0.072) | (0.053) | (0.055) |
| Social-emotional skill (*n* = 1200) | -0.233*** | -0.156*** | -0.304*** | -0.106 | -0.105 | -0.009 | -0.052 | 0.026 |
|  | (0.057) | (0.056) | (0.070) | (0.074) | (0.071) | (0.068) | (0.056) | (0.057) |
| Total child skill factor (*N* = 1200) | -0.093* | -0.015 | -0.139* | 0.028 | 0.035 | 0.210*** | 0.089* | 0.167*** |
|  | (0.047) | (0.046) | (0.073) | (0.061) | (0.062) | (0.070) | (0.046) | (0.047) |
| Material investment factor | -0.003 | 0.059 | -0.000 | 0.089* | 0.070 | 0.334*** | 0.142*** | 0.204*** |
|  | (0.047) | (0.046) | (0.072) | (0.046) | (0.049) | (0.074) | (0.047) | (0.047) |
| Time investment factor | 0.152*** | 0.217*** | -0.138 | 0.246*** | 0.218*** | 0.254*** | 0.303*** | 0.368*** |
|  | (0.049) | (0.049) | (0.087) | (0.062) | (0.062) | (0.078) | (0.049) | (0.049) |
| Parenting skill factor | 0.076 | 0.138*** | 0.050 | 0.220*** | 0.203*** | 0.381*** | 0.220*** | 0.282*** |
|  | (0.048) | (0.047) | (0.071) | (0.055) | (0.057) | (0.071) | (0.047) | (0.048) |

Notes: Table examines the robustness of estimated treatment effects for the parenting center intervention to attrition. Each coefficient comes from a different regression of the outcome (left column) on a dummy for the treatment group. Column (4) presents the main results found in the paper. Column (5) shows coefficients from regressions weighted by attrition propensity, estimated using covariates in Table B2 and their interactions with treatment. Columns (3) and (6) show lower and upper bound s for treatments effects estimated using the procedure in Lee (2009). Columns (1), (2), (7), and (8) impute missing outcomes by taking the treatment-arm specific mean and adding or subtracting an amount equal to 0.1 or 0.25 standard deviations of the outcome in that treatment arm. **p* < 0.10, ***p* < 0.05, ****p* < 0.01

**Appendix C. Comparison of Samples of the Center-Based and Home-Based Interventions**

**Table C1.** Descriptive statistics of baseline characteristics in center-based and home-based program

| Variable | Center-based program | Home-based program | *p*-value |
| --- | --- | --- | --- |
| Male (yes = 1) | 0.52 (0.01) | 0.49 (0.02) | 0.737 |
| Age in months | 14.36 (0.15) | 24.45 (0.14) | 0.000 |
| Low birth weight (yes = 1) | 0.04 (0.01) | 0.04 (0.01) | 0.956 |
| Natural birth (yes = 1) | 0.64 (0.02) | 0.69 (0.02) | 0.797 |
| Caregiver’s completed years of schooling | 8.15 (0.11) | 8.55 (0.12) | 0.347 |
| Mother is the primary caregiver (yes = 1) | 0.70 (0.01) | 0.65 (0.03) | 0.001 |
| Household receives social security support (yes = 1) | 0.11 (0.01) | 0.27 (0.02) | 0.000 |

*Note.* The statistics are the sample mean, with the standard error presented in parentheses. The *p*-values account for clustering at the village level.
